# Protective Effects of Tributyrin on Cyclic Heat-Stressed Taihe Silky Fowls: Insights into Oxidative Status, Inflammatory Response, and Mucosal Barrier Function

**DOI:** 10.3390/antiox14121511

**Published:** 2025-12-17

**Authors:** Chuanbin Chen, Mingren Qu, Guanhong Li, Gen Wan, Huimin Liu, Wenyan Zhang, Lanjiao Xu

**Affiliations:** Jiangxi Province Key Laboratory of Animal Nutrition and Feed, College of Animal Science and Technology, Jiangxi Agricultural University, Nanchang 330045, China; chenchuanb8297@163.com (C.C.); quingren@jxau.edu.cn (M.Q.); liguanh@163.com (G.L.); wg8138@163.com (G.W.); 15970391947@163.com (H.L.); zhangwenyan9911@163.com (W.Z.)

**Keywords:** tributyrin, heat stress, oxidative status, mucosal barrier function, Taihe silky fowls

## Abstract

This study examined the protective impact of tributyrin on heat-stressed Taihe silky fowls, providing insight into oxidative status, inflammatory response, and mucosal barrier function. Three hundred chicks were randomly assigned to 6 treatments: control (CON, 24 ± 1 °C) fed with basal diet and 5 heat stress (HS) treatments (34 ± 1 °C for 8 h/d) fed with basal diet containing 0, 0.04, 0.08, 0.16, and 0.32% tributyrin. Heat stress elevated serum malondialdehyde (MDA), tumor necrosis factor α (TNF-α), interleukin 1β (IL-1β), D-lactate, and diamine oxidase levels, and decreased total antioxidant capacity (T-AOC) and superoxide dismutase (SOD), glutathione peroxidase (GSH-Px), interleukin 4 (IL-4), interleukin 10 (IL-10), and immunoglobulin G (IgG) levels (*p* < 0.05). Compared with HS treatment, tributyrin reversed these serum changes (*p* < 0.05). Moreover, HS elevated jejunal and ileal MDA content and *IL-1β* mRNA abundance, decreased GSH-Px activity, villus height (VH), VH: crypt depth ratio, and mRNA abundance of *IL-10*, *occludin*, and *zonula occludens-1* (*ZO-1*), and decreased cecal butyrate content (*p* < 0.05). Compared with HS treatment, tributyrin reduced jejunal and ileal MDA content and *IL-1β* mRNA abundance, increased GSH-Px activity, VH, and mRNA abundance of *IL-4*, *IL-10*, *occludin*, and *ZO-1*, and increased cecal butyrate content (*p* < 0.05). In conclusion, tributyrin enhanced antioxidant capacity, attenuated inflammatory responses, increased cecal butyrate content, and improved intestinal morphology and mucosal barrier function in cyclic heat-stressed Taihe silky fowls.

## 1. Introduction

With global climate warming and the increasing density of intensive livestock farming, poultry are generally subjected to heat stress [1]. Heat stress adversely impacts poultry health and production, causing economic losses in the industry [2]. Numerous studies have shown that heat stress can trigger various physiological disorders in poultry, including endocrine function disorders, immunosuppression, oxidative stress, and intestinal dysfunction [3,4,5].

The small intestine is essential for nutrient digestion and absorption, while also maintaining the mucosal barrier function and regulating signal transduction [6]. However, the small intestine is prone to being affected by heat stress [7]. Heat stress could weaken the transmembrane barrier function of intestinal epithelial cells in broilers and increase intestinal permeability, thereby damaging intestinal integrity [8,9]. The disruption of the intestinal epithelial barrier integrity can allow toxic substances to invade the intestinal mucosa and trigger an increase in inflammatory cytokines, thereby leading to an imbalance in immune function [10]. Numerous research studies have reported strategies for alleviating heat stress, in which dietary supplementation additives are considered an effective solution [11,12,13,14]. Butyrate has attracted widespread attention because of its nutritional and physiological functions [15].

Butyrate is a short-chain fatty acid that can be rapidly absorbed by epithelial cells through the action of butyrate transporters, providing energy for the intestinal epithelium [16]. Nevertheless, the unpleasant odor and rapid metabolism of butyrate or sodium butyrate have restricted its use in poultry production [17]. Tributyrin is a butyrate prodrug formed through the esterification of one molecule of glycerol with three molecules of butyrate [18]. In the intestines of animals, tributyrin decomposes into butyrate, which is rapidly absorbed and utilized by intestinal epithelial cells [14]. It reported that tributyrin plays a crucial role in regulating immune responses, antioxidant capacity, and anti-inflammatory effects [19,20,21]. Li et al. (2015) also demonstrated that tributyrin improved antioxidant capacity and decrease the secretion of pro-inflammatory cytokines in lipopolysaccharide (LPS)-challenged chickens [22]. Our previous research found that tributyrin improved growth performance and meat quality in heat-stressed Taihe silky fowls [23]. Furthermore, compared to commercial broiler chickens, Taihe silky fowls have poor heat resistance due to their unique filamentous feathers and black skin, which hinder heat dissipation [24,25]. To date, the alleviating effect of dietary tributyrin on intestinal damage in heat-stressed broilers is still unclear. Intestinal damage was highly related to inflammation response and oxidative status [26].

Accordingly, we selected the native Taihe silky fowls (*Gallus domesticus*) as representative models to examine the protective impact of tributyrin on oxidative status, inflammatory responses, and mucosal barrier function in heat-stressed Taihe silky fowls, and to provide practical nutritional strategies for alleviating intestinal damage.

## 2. Materials and Methods

### 2.1. Experiment Design

Four hundred 1-day-old Taihe silky fowls were obtained from a commercial hatchery and provided with a standard commercial diet (Taihe County, Jiangxi Province, China). At 120 days of age, 300 birds (average BW = 915.29 ± 7.99 g) were assigned to 6 dietary treatments, each of which was replicated as five cages with ten broilers per cage. Control treatment (CON, basal diet): birds were raised in a thermoneutral temperature (24 ± 1 °C); heat stress treatment (HS, basal diet): birds were subjected to cyclic heat stress (34 ± 1 °C from 9:30 to 17:30 and 24 ± 1 °C for the remainder of the time); HS + tributyrin treatments (HST1, HST2, HST3, and HST4): birds received the basal diet supplemented with 0.04%, 0.08%, 0.16%, and 0.32% tributyrin (ProPhorce SR 130, butyrate content ≥ 51.1%, Perstorp Waspik B.V., Waspik, The Netherlands) and were reared under cyclic heat stress (CHS) conditions. The dose selection referred to the standards of the previous studies [22,27]. Birds were housed in three-tier wire cages (length = 125 cm, width = 65 cm, and height = 50 cm). As recommended by the Feeding Standard of Chicken, China (NY/T 33-2004), the basal diet (Table 1) was created. The 28-day trial was conducted with birds provided feed and water ad libitum throughout. Additionally, two birds per cage were chosen at random on days 1, 7, 14, 21, and 28, and an electronic thermometer was used to recorded their rectal temperature.

### 2.2. Sample Collection

A bird was randomly chosen from each cage after a 12 h fast, after 28 days of heat exposure. Blood samples were taken from the wing vein and then centrifuged (3500 rpm, 10 min, at 4 °C) to harvest serum. The birds were then euthanized using cervical dislocation followed by exsanguination. The entire gastrointestinal tract was immediately excised, and 2 cm jejunal and ileal mid-sections were separated and preserved in 4% (*w*/*v*) paraformaldehyde for morphology analysis. Mucosal samples from the jejunum and ileum were scraped with a sterile microscope slide. Additionally, cecal digesta was collected. The serum and intestinal samples were stored at −80 °C.

### 2.3. Serum Indicators

According to the manufacturers’ instructions, the levels of total antioxidant capacity (T-AOC, No. A015-2), glutathione peroxidase (GSH-Px, No. A005-1), superoxide dismutase (SOD, No. A001-3), and malondialdehyde (MDA, No. A003-1) were measured with commercial kits (Nanjing Jiancheng Institute of Biotechnology, Nanjing, China). In addition, ELISA kits (Shanghai Enzyme-linked Biotechnology, Shanghai, China) were used to detect the levels of D-lactate (No. ml08770), diamine oxidase (DAO, No. ml036981), immunoglobulins (IgA (No. ml002792), IgG (No. ml042771), and IgM (No. ml890233)) and inflammatory cytokines (TNF-α (No. ml002790), IL-1β (No. ml059835), IL-6 (No. ml059839), IL-4 (No. ml059838), and IL-10 (No. ml059830)). These ELISA kits are only used for the determination of relevant indicators in chicken samples, and the wavelength was 450 nm.

### 2.4. Intestinal Morphology

The samples of jejunum and ileum were preserved in 4% paraformaldehyde. Following a series of graded ethanol dehydration, they were embedded in paraffin and cut with a microtome to a 4 μm thickness. After staining the section for two minutes with hematoxylin and forty seconds with eosin, it was dehydrated and mounted. After that, the stained section was examined and photographed under an optical microscope to assess the morphological changes in the intestine. Using ImageJ software (version 1.54), the villus height (VH) and crypt depth (CD) were determined, and the VH/CD was computed.

### 2.5. Intestinal Mucosa Oxidative Status

The samples of jejunal and ileal mucosa, each weighing 1 g, were carefully homogenized with 9 mL of PBS. Then, the homogenate was centrifuged (3000 rpm, 10 min, at 4 °C) to collect the supernatant for further analysis. Following this, commercial assay kits from the Nanjing Jiancheng Institute were used to measure the levels of antioxidant indicators (T-AOC, GSH-Px, SOD, and MDA) and total protein.

### 2.6. Real-Time qPCR Assay

The Ultrapure RNA kit was used to extract RNA from the jejunum and ileum. The expression levels of related genes were determined using the real-time qPCR technique with the specific primers presented in Table 2. The Real-time qPCR was conducted on a QuantStudio^TM^ 5 Real-Time PCR instrument equipped with a 384-well block (Thermo Scientific, Waltham, MA, USA). In our study, the 10 µL qPCR reaction comprised 5 µL 2 × SuperStar Blue Universal SYBR Master Mix, 0.2 µL each of forward and reverse primers, 1 µL template cDNA, and 3.6 µL ddH_2_O. The above kits were purchased from Kangwei Century Biotechnology (Taizhou, China). The thermal cycling conditions are shown in Table 3. The mRNA abundances were calculated as per the 2^−∆∆Ct^ method.

### 2.7. Short Chain Fatty Acids (SCFAs) in Cecal Chyme

The SCFAs content in cecal chyme was measured as per a method previously described [28]. Briefly, 0.5 g of chyme was homogenized with 3 mL of distilled water and then centrifuged (10,000 rpm, 10 min, at 4 °C) to harvest supernatant, which was combined with 25% metaphosphate (*w*/*v*, 1:5) and centrifuged again under the same conditions. Finally, 1 mL of the supernatant was placed in a screw-cap vial for detection. A GC-8860 gas chromatograph (Agilent, Santa Clara, CA, USA) equipped with a 30 m × 0.25 mm × 0.25 μm capillary column (DB-FastFAME, Agilent) and a flame ionization detector were utilized to analyze the samples. The SCFAs content in each sample was calculated by integrating the peak areas of the standard solution and the sample for comparison.

### 2.8. Statistical Analysis

All experimental data were analyzed using SPSS version 25.0 (Chicago, IL, USA). We utilized one-way ANOVA followed by Tukey’s multiple range test to assess differences among treatments. Results are presented as means ± SEM, with significance defined as *p* < 0.05. The analyses were conducted with the cage as the experimental unit (*n* = 5).

## 3. Results

### 3.1. Rectal Temperature

As seen in Table 4, the rectal temperature of Taihe silky fowls in the HS treatment was higher than that in the CON treatment after heat exposure on days 1, 7, 14, 21, and 28 of heat exposure (*p* < 0.05).

### 3.2. Serum Antioxidant Parameters

**Figure 1:**
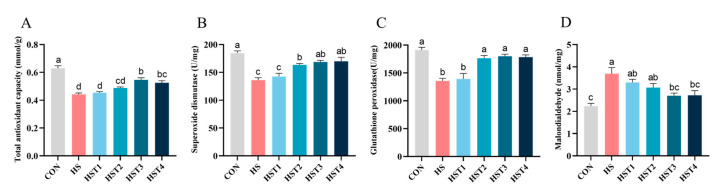
Effect of tributyrin on serum antioxidant parameters in cyclic heat-stressed Taihe silky fowls. (**A**) Total antioxidant capacity, (**B**) superoxide dismutase, (**C**) glutathione peroxidase, and (**D**) malondialdehyde. CON group = basal diet + thermoneutral temperature (24 ± 1 °C); HS group = basal diet + cyclic heat stress (CHS) conditions (34 ± 1 °C from 9: 30 to 17: 30 and 24 ± 1 °C for the rest of the time); HS + tributyrin groups (HST1, HST2, HST3, and HST4), basal diet + 0.04%, 0.08%, 0.16%, and 0.32% tributyrin + CHS conditions. ^a–d^ Groups without common letters differ significantly (*p* < 0.05). *n* = 5.

The effect of tributyrin on serum antioxidant parameters in cyclic heat-stressed Taihe silky fowls is exhibited in Figure 1. Relative to the CON treatment, heat stress significantly decreased serum T-AOC, SOD, and GSH-Px activities and concurrently elevated MDA concentration (*p* < 0.05). Compared with HS treatment, dietary supplementation with 0.08%, 0.16%, and 0.32% tributyrin significantly elevated serum SOD and GSH-Px activities (*p* < 0.05); supplementation with 0.16% and 0.32% tributyrin significantly increased serum T-AOC activity and decreased MDA content in cyclic heat-stressed Taihe silky fowls (*p* < 0.05).

### 3.3. Serum Immunoglobulins

**Figure 2:**
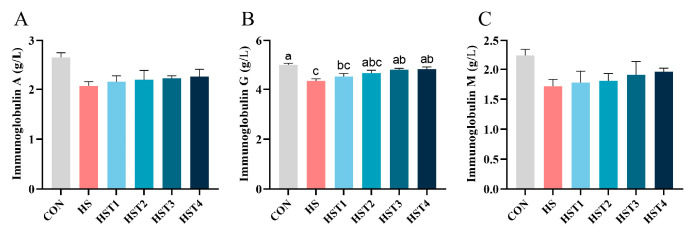
Effect of tributyrin on serum immunoglobulins in cyclic heat-stressed Taihe silky fowls. (**A**) Immunoglobulin A, (**B**) immunoglobulin G, and (**C**) immunoglobulin M. CON group = basal diet + thermoneutral temperature (24 ± 1 °C); HS group = basal diet + cyclic heat stress (CHS) conditions (34 ± 1 °C from 9: 30 to 17: 30 and 24 ± 1 °C for the rest of the time); HS + tributyrin groups (HST1, HST2, HST3, and HST4), basal diet + 0.04%, 0.08%, 0.16%, and 0.32% tributyrin + CHS conditions. ^a–c^ Groups without common letters differ significantly (*p* < 0.05). *n* = 5.

Serum immunoglobulin concentrations were quantified to evaluate the impact of tributyrin on the immune function in the serum of Taihe silky fowls under CHS conditions (Figure 2). Heat stress reduced serum IgG concentration relative to the CON treatment (*p* < 0.05). Compared with HS treatment, dietary addition of 0.16% and 0.32% tributyrin markedly elevated serum IgG content in cyclic heat-stressed Taihe silky fowls (*p* < 0.05). No significant difference was observed in serum IgA and IgM levels among treatments (*p* > 0.05).

### 3.4. Serum Inflammatory Cytokines

**Figure 3:**
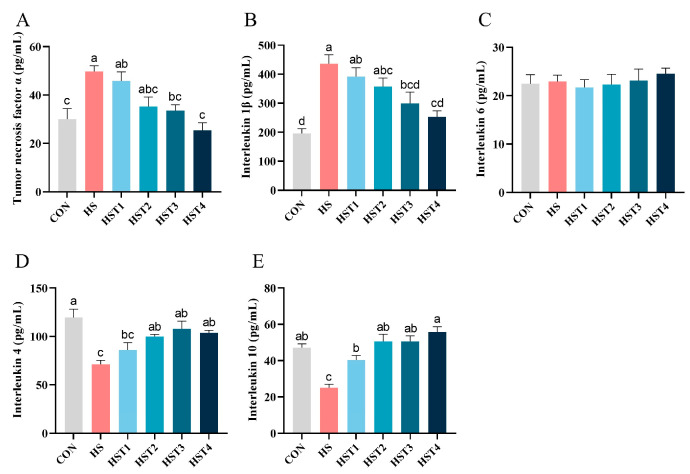
Effect of tributyrin on serum cytokines in cyclic heat-stressed Taihe silky fowls. (**A**) Tumor necrosis factor α, (**B**) interleukin 1β, (**C**) interleukin 6, (**D**) interleukin-4, and (**E**) interleukin-10. CON group = basal diet + thermoneutral temperature (24 ± 1 °C); HS group = basal diet + cyclic heat stress (CHS) conditions (34 ± 1 °C from 9: 30 to 17: 30 and 24 ± 1 °C for the rest of the time); HS + tributyrin groups (HST1, HST2, HST3, and HST4), basal diet + 0.04%, 0.08%, 0.16%, and 0.32% tributyrin + CHS conditions. ^a–d^ Groups without common letters differ significantly (*p* < 0.05). *n* = 5.

Relative to the CON treatment, heat stress elevated serum TNF-α and IL-1β concentrations, as well as reducing IL-4 and IL-10 concentrations (*p* < 0.05, Figure 3). Compared with HS treatment, dietary supplementation with 0.04%, 0.08%, 0.16%, and 0.32% tributyrin significantly increased serum IL-10 concentration (*p* < 0.05); supplementation with 0.08%, 0.16 and 0.32% tributyrin significantly enhanced serum IL-4 concentration (*p* < 0.05); supplementation with 0.16% and 0.32% tributyrin significantly reduced serum TNF-α and IL-1β concentrations (*p* < 0.05) in cyclic heat-stressed Taihe silky fowls. However, no significant difference in serum IL-6 was observed among treatments (*p* > 0.05).

### 3.5. Serum D-Lactate and DAO

**Figure 4:**
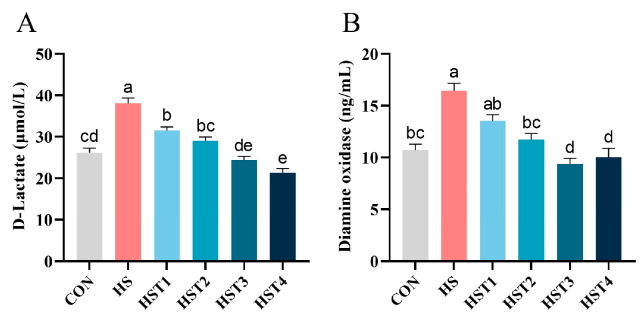
Effect of tributyrin on serum (**A**) D-lactate and (**B**) diamine oxidase in cyclic heat-stressed Taihe silky fowls. CON group = basal diet + thermoneutral temperature (24 ± 1 °C); HS group = basal diet + cyclic heat stress (CHS) conditions (34 ± 1 °C from 9: 30 to 17: 30 and 24 ± 1 °C for the rest of the time); HS + tributyrin groups (HST1, HST2, HST3, and HST4), basal diet + 0.04%, 0.08%, 0.16%, and 0.32% tributyrin + CHS conditions. ^a–e^ Groups without common letters differ significantly (*p* < 0.05). *n* = 5.

The concentrations of serum D-lactate and DAO were measured to assess the impact of tributyrin on intestinal permeability of Taihe silky fowls under CHS conditions (Figure 4). Heat stress elevated serum D-lactate and DAO concentrations relative to the CON treatment (*p* < 0.05). Compared with HS treatment, dietary supplementation with 0.04%, 0.08%, 0.16%, and 0.32% tributyrin markedly reduced serum D-lactate concentration (*p* < 0.05); supplementation with 0.08%, 0.16%, and 0.32% tributyrin significantly decreased serum DAO activity in cyclic heat-stressed Taihe silky fowls (*p* < 0.05).

### 3.6. Intestinal Morphology

**Figure 5:**
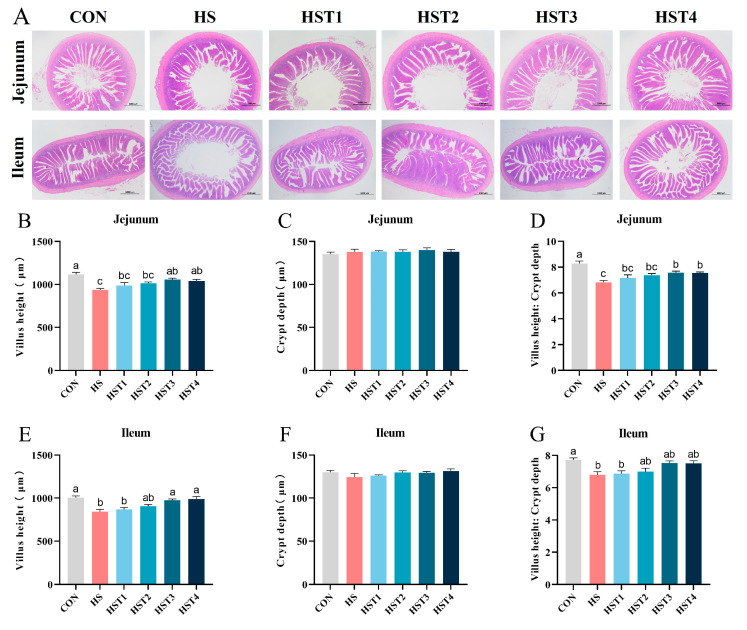
Effect of tributyrin on intestinal morphology in cyclic heat-stressed Taihe silky fowls. (**A**) A representative hematoxylin and eosin (H&E) image of the jejunum and ileum was observed at a magnification of 20×. Villus height of jejunum (**B**) and ileum (**E**). Crypt depth of jejunum (**C**) and ileum (**F**). Villus height: Crypt depth of jejunum (**D**) and ileum (**G**). CON group = basal diet + thermoneutral temperature (24 ± 1 °C); HS group = basal diet + cyclic heat stress (CHS) conditions (34 ± 1 °C from 9: 30 to 17: 30 and 24 ± 1 °C for the rest of the time); HS + tributyrin groups (HST1, HST2, HST3, and HST4), basal diet + 0.04%, 0.08%, 0.16%, and 0.32% tributyrin + CHS conditions. ^a–c^ Groups without common letters differ significantly (*p* < 0.05). *n* = 5.

Figure 5 showed that heat stress reduced VH and VH/CD in the jejunum and ileum relative to the CON treatment (*p* < 0.05). Compared with HS treatment, dietary supplementation with 0.16% and 0.32% tributyrin significantly elevated jejunal and ileal VH, as well as jejunal VH/CD in cyclic heat-stressed Taihe silky fowls (*p* < 0.05).

### 3.7. Intestinal Mucosa Antioxidant Parameters

**Figure 6:**
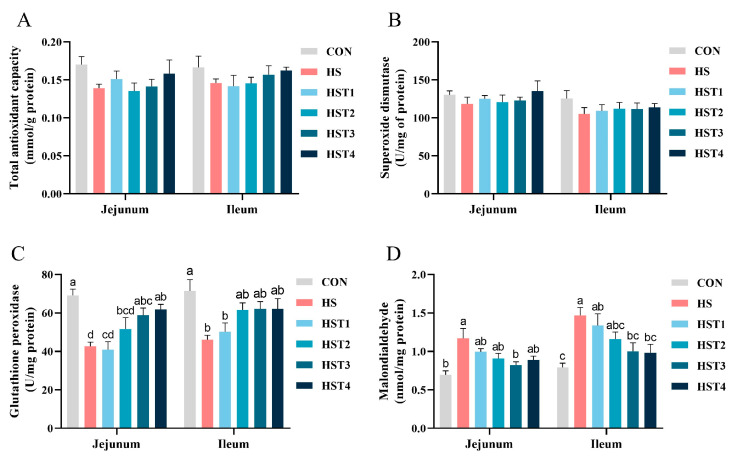
Effect of tributyrin on the antioxidant parameters in both jejunal and ileal mucosa in cyclic heat-stressed Taihe silky fowls. (**A**) Total antioxidant capacity, (**B**) superoxide dismutase, (**C**) glutathione peroxidase, and (**D**) malondialdehyde. CON group = basal diet + thermoneutral temperature (24 ± 1 °C); HS group = basal diet + cyclic heat stress (CHS) conditions (34 ± 1 °C from 9: 30 to 17: 30 and 24 ± 1 °C for the rest of the time); HS + tributyrin groups (HST1, HST2, HST3, and HST4), basal diet + 0.04%, 0.08%, 0.16%, and 0.32% tributyrin + CHS conditions. ^a–d^ Groups without common letters differ significantly (*p* < 0.05). *n* = 5.

Figure 6 summarizes the effects of tributyrin on antioxidant parameters in the intestinal mucosa of Taihe silky fowls under CHS conditions. Compared with the CON treatment, heat stress markedly decreased GSH-Px activity and increased MDA concentration in both jejunal and ileal mucosa (*p* < 0.05). Compared with HS treatment, dietary supplementation with 0.16% and 0.32% tributyrin significantly increased GSH-Px activity in the jejunal mucosa and decreased MDA content in the ileal mucosa (*p* < 0.05); supplementation with 0.16% tributyrin also decreased jejunal mucosal MDA content (*p* < 0.05).

### 3.8. Relative mRNA Expression of Jejunal and Ileal Mucosa

**Figure 7:**
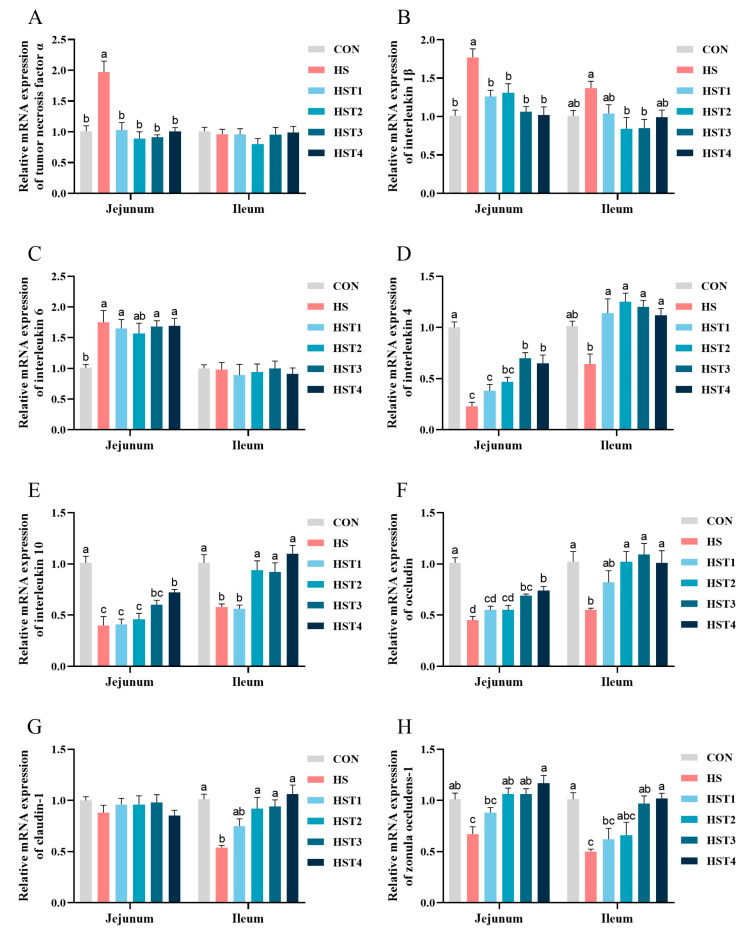
Effect of tributyrin on the mRNA abundance of jejunal and ileal mucosa in cyclic heat-stressed Taihe silky fowls. (**A**) Tumor necrosis factor α, (**B**) interleukin 1β, (**C**) interleukin 6, (**D**) interleukin-4, (**E**) interleukin-10, (**F**) occludin, (**G**) claudin-1, and (**H**) zonula occludens-1. CON group = basal diet + thermoneutral temperature (24 ± 1 °C); HS group = basal diet + cyclic heat stress (CHS) conditions (34 ± 1 °C from 9: 30 to 17: 30 and 24 ± 1 °C for the rest of the time); HS + tributyrin groups (HST1, HST2, HST3, and HST4), basal diet + 0.04%, 0.08%, 0.16%, and 0.32% tributyrin + CHS conditions. ^a–d^ Groups without common letters differ significantly (*p* < 0.05). *n* = 5.

Compared with the CON treatment, heat stress significantly elevated the mRNA abundances of *TNF-α*, *IL-1β*, and *IL-6* in the jejunal mucosa (*p* < 0.05, Figure 7). Compared with HS treatment, dietary supplementation with 0.04%, 0.08%, 0.16%, and 0.32% tributyrin significantly decreased the mRNA abundances of *TNF-α* and *IL-1β* in the jejunal mucosa (*p* < 0.05); supplementation with 0.08% and 0.16% tributyrin decreased ileal mucosal *IL-1β* mRNA abundance (*p* < 0.05). Additionally, heat stress significantly decreased the mRNA abundances of *IL-4*, *IL-10*, *occludin*, and *ZO-1* in the jejunal mucosa (*p* < 0.05), as well as decreased the mRNA abundances of *IL-10*, *occludin*, *claudin-1*, and *ZO-1* in the ileal mucosa (*p* < 0.05). Compared with HS treatment, dietary supplementation with 0.04%, 0.08%, 0.16%, and 0.32% tributyrin significantly increased ileal mucosal *IL-4* mRNA abundance (*p* < 0.05). Additionally, supplementation with 0.08%, 0.16%, and 0.32% tributyrin significantly elevated jejunal mucosal *ZO-1* and ileal mucosal *IL-10*, *occludin*, and *claudin-1* mRNA abundances (*p* < 0.05); supplementation with 0.16% and 0.32% tributyrin also increased jejunal mucosal *IL-4* and *occludin* mRNA abundances together with ileal mucosal *ZO-1* mRNA abundance (*p* < 0.05); and supplementation with 0.32% tributyrin significantly raised jejunal mucosal *IL-10* mRNA abundance (*p* < 0.05).

### 3.9. SCFAs in Cecal Chyme

**Figure 8:**
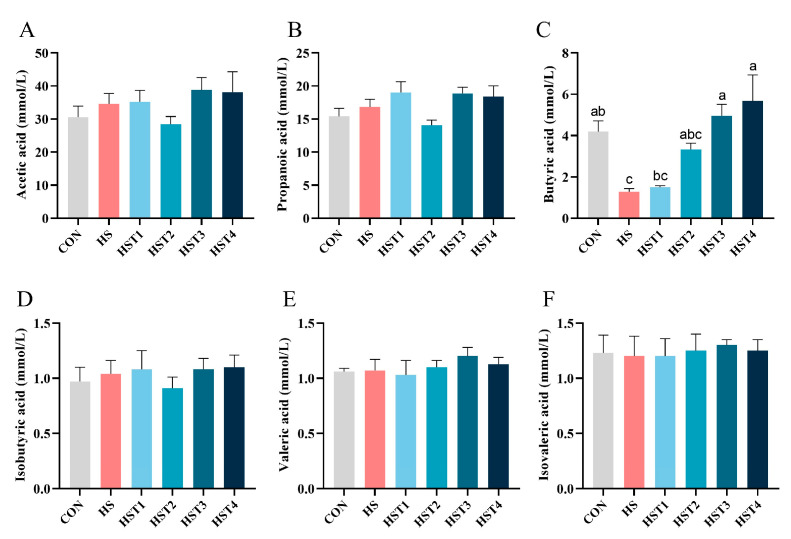
Effect of tributyrin on SCFAs content of cecal digesta in cyclic heat-stressed Taihe silky fowls. (**A**) acetic acid, (**B**) propanoic acid, (**C**) butyric acid, (**D**) isobutyric acid, (**E**) valeric acid, and (**F**) isovaleric acid. CON group = basal diet + thermoneutral temperature (24 ± 1 °C); HS group = basal diet + cyclic heat stress (CHS) conditions (34 ± 1 °C from 9: 30 to 17: 30 and 24 ± 1 °C for the rest of the time); HS + tributyrin groups (HST1, HST2, HST3, and HST4), basal diet + 0.04%, 0.08%, 0.16%, and 0.32% tributyrin + CHS conditions. ^a–c^ Groups without common letters differ significantly (*p* < 0.05). n = 5.

The concentration of SCFAs (acetic acid, propanoic acid, butyric acid, isobutyric acid, valeric acid, and isovaleric acid) among the treatments was analyzed and exhibited in Figure 8. Compared with the CON treatment, the HS treatment lowered cecal butyric acid concentration (*p* < 0.05). Compared with HS treatment, dietary addition of 0.16% and 0.32% tributyrin increased cecal butyric acid concentration in cyclic heat-stressed Taihe silky fowls (*p* < 0.05).

## 4. Discussion

The long-term exposure to elevated temperatures renders poultry particularly susceptible to HS due to their higher metabolic rate and lack of sweat glands [2]. Ambient temperatures above the broiler’s thermoneutral zone (16~26 °C) impose HS, rapidly manifesting as hyperthermia and a spectrum of physiological disturbances [29]. Throughout the experiment, Taihe silky fowls exposed to HS exhibited an increase in rectal temperature at all five sampling time points, confirming the successful establishment of the heat-stressed model, consistent with previous research findings [30].

Generally, HS can lead to oxidative stress, particularly when the antioxidant defense system is dysregulated [31]. Research has demonstrated that HS can generate excessive free radicals, cause lipid peroxidation, and disrupt the redox balance, ultimately resulting in oxidative damage to tissue [14,26]. Our findings indicated that HS elevated serum and mucosal (jejunum and ileum) MDA concentrations, while simultaneously decreasing serum T-AOC, SOD, and GSH-Px levels, as well as mucosal GSH-Px activities in the jejunum and ileum. Recent studies have suggested that dietary tributyrin acts as an antioxidant by boosting GSH-Px and SOD activity, while inhibiting lipid peroxidation in animals [22,32,33]. Similarly, supplementation with tributyrin led to increases in serum T-AOC, SOD, and GSH-Px levels, as well as elevated GSH-Px activities in the mucosal tissues of the jejunum and ileum. At the same time, tributyrin reduced MDA content, supporting its role as an antioxidant found in previous research. Our results suggested that tributyrin can improve the redox status of intestinal mucosa in heat-stressed Taihe silky fowls by enhancing the activity of key antioxidant enzymes.

Inflammatory responses frequently accompany oxidative stress [34]. The levels of inflammatory cytokines can serve as indirect indicators of oxidative damage [35]. In our study, HS elevated the concentrations of serum TNF-α and IL-1β, while decreasing the levels of IL-4 and IL-10. It has been reported that butyric acid can directly suppress macrophage-mediated anti-inflammatory and modulate immune responses [36]. Our research found that dietary supplementation with tributyrin decreased the concentrations of serum IL-1β and TNF-α, while increasing the levels of IL-4, IL-10, and IgG in cyclic heat-stressed Taihe silky fowls. Zou et al. (2019) also reported that sodium butyrate lowered serum levels of TNF-α and IL-1β, while raising IL-10 in chickens [37]. Tang and Chen (2016) found that γ-aminobutyric acid increased plasma concentrations of IgA, IgG, and IgM in heat-stressed broilers [38]. Moreover, tributyrin down-regulated the mRNA abundance of *IL-1β* in the jejunal and ileal mucosa, while up-regulating the mRNA abundances of *IL-4* and *IL-10* in the same segments. Thus, dietary tributyrin supplementation could enhance immunity and alleviate inflammation in cyclic heat-stressed Taihe silky fowls.

Acting as a vital protective barrier, the intestinal mucosa not only aids in nutrient digestion and absorption but also serves as an important indicator for assessing the integrity of intestinal development and function [39]. Numerous studies have shown that HS induces lipid peroxidation damage, which impairs the intestinal mucosa, causing the VH to become shorter and the CD to become shallower [6,13]. As expected, HS results in a decrease in VH in the jejunum and a reduced VH/CD. Dietary supplementation with tributyrin has been shown to enhance intestinal architecture by promoting mucosal repair and improving intestinal morphology, which in turn increases nutrient absorption efficiency [27]. It is undoubtedly that tributyrin supplementation can improve intestinal morphology of chickens under normal conditions [40]. Furthermore, previous studies have reported that adding butyrate and its derivatives, such as γ-aminobutyric acid and tributyrin, to the diet can effectively reduce damage to the intestinal morphology of poultry following post-*Eimeria* challenges or HS [33,41,42]. Our data showed that adding tributyrin to the feed effectively improved intestinal morphology in Taihe silky fowls under CHS conditions.

The DAO is an intracellular enzyme mainly produced by epithelial cells located at the tips of intestinal villi [43]. D-lactate is primarily generated by gut bacteria through the fermentation of carbohydrates [44]. Elevated levels of serum DAO activity and D-lactate suggest increased intestinal permeability, which serves as a valuable indicator for assessing the integrity of the intestinal barrier [45]. In our study, we observed that HS led to increases in serum DAO and D-lactate levels, which aligns with previous findings [26,46].

The tight junctions (**TJs**) of the gut epithelium, which consist of the adaptor protein ZO-1 and the transmembrane proteins occludin and claudins, are indispensable for preserving mucosa barrier function [47]. Cheng et al. (2019) demonstrated that HS reduced the mRNA abundances of *occludin* in jejunal mucosa, as well as *ZO-1* and occludin in the ileal mucosa of chickens [30]. Deng et al. (2023) also observed that HS decreased the mRNA abundance of *occludin* and *ZO-1* the ileal mucosa of broilers [48]. In this study, we found that HS reduced the mRNA abundances of *occludin* and *ZO-1* in both the jejunal and ileal mucosa, and it also suppressed *claudin-1* mRNA in the ileum. These findings suggest that HS may disrupt intestinal barrier integrity, which could explain the concurrent increase in serum DAO activity and D-lactate concentration. Tributyrin has been shown to positively influence mucosal barrier function in weaned pigs [32]. Additionally, tributyrin can enhance the expression of TJs in broilers affected by *Eimeria maxima* induced by coccidiosis [49]. In our study, dietary tributyrin decreased serum D-lactate level and DAO activity. Moreover, tributyrin improved mucosa barrier integrity by up-regulating the mRNA abundance of *occludin* and *ZO-1* in the jejunal and ileal mucosa, as well as *claudin-1* in the ileal mucosa. These results indicated that tributyrin could effectively protect against HS-induced mucosal barrier dysfunction in broilers.

The SCFAs play an important role in maintaining normal pH values in the intestine, promoting the proliferation of beneficial microbiota, suppressing pathogen colonization, and thereby reinforcing epithelial barrier integrity [50]. In poultry, the concentration of SCFAs in the cecum increases consistently with higher dietary levels of tributyrin or sodium butyrate [40,51]. Furthermore, under CHS conditions, the levels of butyric acid in the cecum significantly rise, with high doses of tributyrin being the most effective in this study. This aligned with tributyrin’s role in improving intestinal morphology in the present study. These findings suggested that tributyrin might enhance intestinal development by increasing the content of butyric acid.

Gut microbiota plays a pivotal role in maintaining intestinal homeostasis [52]. Previous work showed that tributyrin elevated cecal *Lactobacillus* abundance in broilers kept under thermoneutral conditions [40]. The *Lactobacillus* secretes various antibacterial substances and mucins that reinforce the epithelial barrier, exclude pathogens, and promote the proliferation of the beneficial microbial community [53]. Our earlier study demonstrated that dietary tributyrin increased the relative abundance of cecal *Rikenellaceae_RC9_gut_group* and tended to raise (*p* = 0.096) *Lactobacillus*, while improving growth performance in cyclic heat-stressed Taihe silky fowls [23]. The *Rikenellaceae_RC9_gut_group* is a fiber-degrading, SCFAs-producing bacterial group within the *Bacteroidota* phylum that contributes to gut health [54]. Thus, tributyrin might boost the content of butyric acid by regulating the abundance of microbiota.

In this study, tributyrin supplementation decreased MDA concentrations and increased GSH-Px activity, accompanied by reduced *TNF-α* and *IL-1β* mRNA expression and diminished inflammatory cytokine release. However, the major limitations of the present study are that the key signaling pathways, such as nuclear factor erythroid 2-related factor 2 (Nrf2) and nuclear factor κB (NF-κB), were not validated. In the lipopolysaccharide-induced inflammation model, supplementation with butyrate reduced inflammatory markers, including NF-κB and IL-1β [55]. Moreover, butyrate also increased the mRNA expression of *Nrf2* in the colon piglets [56]. Similarly, sodium butyrate bolstered antioxidant defenses and suppressed inflammatory markers (IL-6, IL-1β, TNF-α) by activating Nrf2 while inhibiting the NF-κB pathway, thereby protecting bovine mammary epithelial cells from LPS-induced injury [57]. As a butyrate precursor, tributyrin has likewise been shown to suppress inflammation and elevate antioxidant capacity [22,58]. Thus, whether tributyrate alleviates the HS-induced oxidative damage and inflammation by modulating the Nrf2 and NF-κB pathways still requires further in vivo and in vitro studies to validate. In addition, no significant differences in IgA, IgM, IL-6, and SCFAs (acetic acid, propanoic acid, butyric acid, isobutyric acid, valeric acid, and isovaleric acid) between the different concentrations of tributyrin in this study. This aspect could be explained based on a relatively insufficient number of observations, as well as the fact that heat stress lasted only 28 days. Therefore, enhancing the sample size of the experiment and extending the trial period. These are essential for future research.

## 5. Conclusions

**Figure 9:**
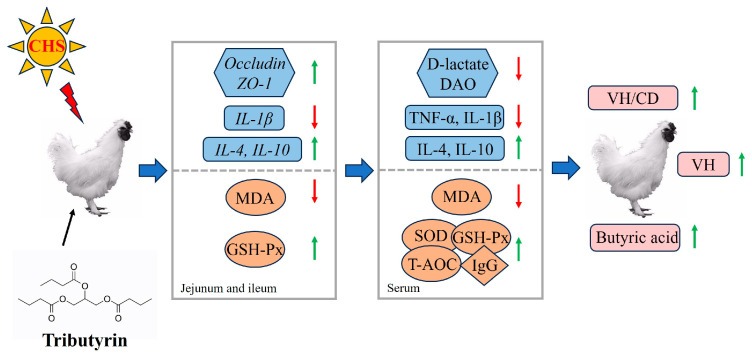
Schematic model illustrating the mechanism of tributyrin alleviates cyclic heat stress-induced inflammation and oxidative damage of Taihe silky fowls. The red color arrow indicated a decrease in the parameters, while the green color arrow represented an increase in the parameters.

In summary, the conclusion could be reached that, as shown in Figure 9, dietary tributyrin increased antioxidant capacity and mitigated inflammatory responses in heat-stressed Taihe silky fowls. Additionally, tributyrin effectively alleviated intestinal damage by increasing cecal butyrate content and improving both intestinal morphology and mucosal barrier function.

## Figures and Tables

**Table 1:** Composition and nutrient contents of basal diets.

Ingredients (g/kg)		Nutritional Composition	
Corn	676	Metabolizable energy (MJ/kg)	12.97
Soybean meal	220	Crude protein (g/kg)	161.9
Fish meal	20	Calcium (g/kg)	8.1
Soybean oil	40	Available phosphorus (g/kg)	3.8
Dicalcium phosphate	10	Threonine (g/kg)	6.9
Limestone	11	Lysine (g/kg)	8.6
_DL_-Methionine	0.9	Methionine (g/kg)	3.6
Threonine	0.5	Methionine + cystine (g/kg)	6.5
Salt	3		
Zeolite powder	13.6		
Premix ^1^	5		
Total	1000		

^1^ The premix provided per kilogram of diet: vitamin A: 11250 IU; vitamin D3: 3750 IU; vitamin E: 30 IU; vitamin K: 1.75 mg; thiamin: 2.75 mg; riboflavin: 9.30 mg; calcium pantothenate: 18.75 mg; nicotinamide: 37.50 mg; vitamin B6: 3.75 mg; biotin: 0.15 mg; folic acid: 1.80 mg; vitamin B12: 0.028 mg; choline chloride: 720 mg; ferrous: 80 mg; copper: 8 mg; manganese: 80 mg; zinc: 60 mg; iodine: 0.35 mg; selenium: 0.15 mg.

**Table 2:** Sequences for real-time qPCR PCR primers.

Genes	GenBank Number	Primer Sequences (5′ to 3′ Direction)
*IL-1β*	NM_204524.2	Forward: GCATCAAGGGCTACAAGCTC
		Reverse: GTCCAGGCGGTAGAAGATGA
*IL-4*	NM-001007079.1	Forward: CGGCGATGCTCTGTTATCTG
		Reverse: TAGGAGGCAGATGGTGCAAA
*IL-* *6*	NM_204628.2	Forward: AGGCCGCTCCGAAAGTAATA
		Reverse: GTTCAACCTCTGCTGCCATT
*IL-* *10*	NM_001004414.4	Forward: CTCTGAGCACAGTCGTTTGG
		Reverse: TCGTCTGGTGTTTGCAGTTG
*TNF-* *α*	MF000729.1	Forward: CACAGCTCCGCTCAGAAC
		Reverse: GGGACCACCAGTTTGTTCCT
*Claudin1*	NM_001013611.2	Forward: AAGTGCATGGAGGATGACCA
		Reverse: GAGCCACTCTGTTGCCATAC
*Occludin*	NM_205128.1	Forward: CCTCATCGTCATCCTGCTCT
		Reverse: GGTCCCAGTAGATGTTGGCT
*ZO-1*	XM_046925214.1	Forward: GGAACAACACACGGTGACTC
		Reverse: GCCTCCTTTCAGCACATCAG
*β-actin*	NM_205518.2	Forward: GATTTCGAGCAGGAGATGGC
		Reverse: GCCAATGGTGATGACCTGAC

**Table 3:** Real-time qPCR thermal cycling conditions.

Step	Temperature	Time	Cycle
Pre-denaturation	95 °C	30 s	1
Denaturation	95 °C	10 s	42
Annealing/extension	60 °C	30 s	
Melting curve	Use the default melting curve acquisition program of the instrument		

**Table 4:** Effects of tributyrin on cloacal temperatures in cyclic heat-stressed Taihe silky fowls, °C.

Items		Treatments ^1^						
	CON	HS	HST1	HST2	HST3	HST4	SEM	*p*-Value
1 d	41.76 ^a^	42.92 ^b^	42.76 ^b^	42.82 ^b^	42.78 ^b^	42.68 ^b^	0.078	<0.001
7 d	41.64 ^a^	42.54 ^b^	42.52 ^b^	42.50 ^b^	42.38 ^b^	42.36 ^b^	0.074	<0.001
14 d	41.70 ^a^	42.52 ^b^	42.58 ^b^	42.60 ^b^	42.46 ^b^	42.34 ^b^	0.075	<0.001
21 d	41.68 ^a^	42.52 ^b^	42.62 ^b^	42.44 ^b^	42.46 ^b^	42.24 ^b^	0.075	<0.001
28 d	41.58 ^a^	42.46 ^b^	42.44 ^b^	42.26 ^b^	42.16 ^b^	42.30 ^b^	0.069	<0.001

^1^ CON group = basal diet + thermoneutral temperature (24 ± 1 °C); HS group = basal diet + cyclic heat stress (CHS) conditions (34 ± 1 °C from 9:30 to 17:30 and 24 ± 1 °C for the rest of the time); HS + tributyrin groups (HST1, HST2, HST3, and HST4), basal diet + 0.04%, 0.08%, 0.16%, and 0.32% tributyrin + CHS conditions. Values without common superscripts in the same row differ significantly (*p* < 0.05). *n* = 5. Abbreviation: d, day.

## Data Availability

The original contributions presented in this study are included in the article. Further inquiries can be directed to the corresponding author.

## Abbreviations

The following abbreviations are used in this manuscript:

T-AOC	Total antioxidant capacity
GSH-Px	Glutathione peroxidase
SOD	Superoxide dismutase
DAO	Diamine oxidase
MDA	Malondialdehyde
IgA	Immunoglobulin A
IgG	Immunoglobulin G
IgM	Immunoglobulin M
TNF-α	Tumor necrosis factor α
IL-1β	Interleukin 1β
IL-6	Interleukin 6
IL-4	Interleukin 4
IL-10	Interleukin 10
ZO-1	Zonula occludens-1
VH	Villus height
CD	Crypt depth
